# Severe-Enduring Anorexia Nervosa (SE-AN): a case series

**DOI:** 10.1186/s40337-023-00925-6

**Published:** 2023-11-22

**Authors:** Federica Marcolini, Alessandro Ravaglia, Silvia Tempia Valenta, Giovanna Bosco, Giorgia Marconi, Federica Sanna, Giulia Zilli, Enrico Magrini, Flavia Picone, Diana De Ronchi, Anna Rita Atti

**Affiliations:** 1 Department of Biomedical and Neuromotor Sciences, University of Bologna, Viale Pepoli 5, 40123 Bologna, Italy; 2 Department of Clinical Nutrition, AUSL Bologna, Bologna, Italy; 3 U.O. Cure Primarie, AUSL Area Vasta Romagna, ambito di Rimini, Rimini, Italy

**Keywords:** Anorexia Nervosa, Eating disorders, Chronicity, Severe-enduring, Case series

## Abstract

**Background:**

Anorexia Nervosa (AN) poses significant therapeutic challenges, especially in cases meeting the criteria for Severe and Enduring Anorexia Nervosa (SE-AN). This subset of AN is associated with severe medical complications, frequent use of services, and the highest mortality rate among psychiatric disorders.

**Case presentation:**

In the present case series, 14 patients were selected from those currently or previously taken care of at the Eating Disorders Outpatients Unit of the Maggiore Hospital in Bologna between January 2012 and May 2023. This case series focuses on the effects of the disease, the treatment compliance, and the description of those variables that could help understand the great complexity of the disorder.

**Conclusion:**

This case series highlights the relevant issue of resistance to treatment, as well as medical and psychological complications that mark the life course of SE-AN patients. The chronicity of these disorders is determined by the overlapping of the disorder's ego-syntonic nature, the health system's difficulty in recognizing the problem in its early stages, and the presence of occupational and social impairment.

## Introduction

Anorexia Nervosa (AN) constitutes a complex eating disorder (ED) characterized by low caloric intake, fear of gaining weight, dysfunctional behavior impeding weight gain, and misperceptions about one’s own body shape and weight [1]. The intricate nature of AN can lead to severe difficulties in the treatment, and patients may not necessarily benefit from conventional approaches. Most AN patients reach partial or complete remission only after several years from the development of the first symptoms [2]. Despite substantial intervention efforts, an estimated 20% of AN patients show limited improvements and, over time, become chronic [3, 4]. Despite that, there are very few studies on chronic AN, especially in older populations, probably due to the relatively high drop out rate after a few years of treatment [5, 6].

To delineate the domain of chronic AN, the definition of Severe and Enduring Anorexia Nervosa (SE-AN) had been proposed [7] (Table 1), accompanied by suggested maintenance factors [8]. This definition is useful to describe and analyze the peculiarities of chronic patients, to improve the relatively broad criteria for SE-AN definition and to better understand the clinical development of this ED. The search for a precise terminological framework has shown that the quality of the words used in the definitions, in addition to carrying the risk of stigmatizing patients, can influence the way patients and their families experience ED. Moreover, it can have a significant impact on the perspectives that clinicians have on treatment. For instance, the labels 'chronic' and 'treatment resistant' can both affect clinicians' perspective on a patient's curability or their willingness to engage cooperatively during the treatment. Other terms, such as 'severe and enduring’, 'long-lasting' and similar expressions relating to the severity and duration variation of the disease, may favor a lesser focus on the curability of an individual.

**Table 1:** SE-AN diagnostic criteria, as proposed by Hay et Touyz (2018) [7]

1	A persistent state of dietary restriction, underweight, and overvaluation of weight/shape with functional impairment
2	Duration of > 3 years of anorexia nervosa
3	Exposure to at least two evidence-based treatments appropriately delivered together with a diagnostic assessment and formulation that incorporates an assessment of the person’s ED, health literacy, and stage of change

In the present case series, we aim to provide an overview of the possible associations that exist among the multiple variables, causal or consequential, peculiar to SE-AN. The search for common causal factors, although within a small population of patients with SE-AN, may facilitate a better understanding of the disorder and its main determinants in order to intercept cases that are more likely to develop a long history of illness or severe forms that would necessitate intensive treatment. It could also help to improve and personalize the therapeutic approach towards this specific population, considering that even today the available treatments do not always guarantee a positive outcome. In addition, this case series directs its focus on the effects of the disease, treatment compliance, and the description of variables that could contribute to a comprehensive understanding of the complex nature of the disorder.

## Materials and methods

In the present case series, 14 patients were selected from those currently or previously taken care of at the ED Outpatients Unit of the Maggiore Hospital in Bologna between January 2012 and May 2023. To make the sample more homogeneous, we selected only adult patients (age > 18 years, by Italian law) who fully fit the eligibility criteria for SE-AN, as proposed by Hay and Touyz in 2018 [7].

Information on individual patients was obtained through data collection from several available sources: Electronic Medical Records (EMR), paper records, and other documents available in the psychiatric department.

## Results

### Demographic characteristics

A total of 14 patients were enrolled, all from Bologna and its province, and all with a history of admission to Public Psychiatric Services (100%). Five patients (35.7%) were still in psychiatric service care, while four patients dropped out of services (28.6%). One patient had died. At the time of data collection, four patients (8.6%) were no longer followed within a psychiatric pathway. A summary of the main clinical and social characteristics of the patients is presented in Tables 2 and 3.

**Table 2:** A summary of patients’ main clinical and social characteristics

	Sex	Age	Living with someone	Children	Currently employed	Age of onset	Age at diagnosis	Disease duration (years)	Duration of untreated ED (years)
Pt.1	F	53	Yes (1)	No	No	26	27	> 22	1
Pt.2	F	30	Yes (2)	No	No	16	27	14	11
Pt.3	F	47	Yes (3)	No	X	20	28	> 15	8
Pt.4	F	37	Yes (3)	No	Yes	29	30	8	1
Pt.5	F	38	No	No	Yes	16	36	> 20	20
Pt.6	F	63	Yes (1)	Yes	No	47	49	> 9	2
Pt.7	F	54	Yes (1)	Yes	X	17	51	> 31	34
Pt.8	F	41	Yes (2)	Yes	No	15	30	26	15
Pt.9	F	46	Yes (3)	No	No	31	36	15	4
Pt.10	F	55	Yes (1)	No	No	30	44	> 17	14
Pt.11	F	31	Yes (3)	No	No	17	18	13	1
Pt.12	F	44	Yes (3)	No	No	25	27	> 12	2
Pt.13	F	28	No	No	Yes	18	22	20	4
Pt.14	F	24	Yes (3)	No	No	11	16	23	5

*Pt* patient; (1): Husband/Own Family; (2): Partner; (3): Parents; *X* information not given; *ED* eating disorders

**Table 3:** A summary of patients’ main clinical characteristics

	Diagnosis	Minimum BMI	Internal medicine hospitalization	Psychiatric hospitalization	ED specific setting
Pt.1	AN-BP	13.84	Yes	Yes	Yes
Pt.2	AN-R	14.28	No (4)	No	No (4)
Pt.3	AN-R	9.79	Yes	No	Yes
Pt.4	AN-R	13.5	Yes	No	Yes
Pt.5	AN-BP	13.5	Yes	No	No (4)
Pt.6	AN-R	9.65	Yes	No	No (4)
Pt.7	AN-R	X	No	Yes	Yes
Pt.8	AN-R	14.8	Yes	Yes	No (4)
Pt.9	AN-R	11.34	Yes	No	No (5)
Pt.10	AN-R	12.41	Yes	Yes	Yes
Pt.11	AN-R	9.4	Yes	Yes	Yes
Pt.12	AN-R	7.15	Yes	Yes	Yes
Pt.13	AN-R	13.1	No	No	Yes
Pt.14	AN-R	12	Yes	Yes	Yes

*Pt* patient, *BMI* Body Mass Index, *ED* eating disorders,(4): Refused; (5) Decision in progress; *X* information not given

The patients were all female (100%). The sample had an average age of 42.2 years old (ranging from 24 to 63 years old). Only four out of 14 patients (28.6%) were married and none of them had gone through a divorce. Three patients (21.4%) had at least one child, and only one had more than one. Therefore, the majority of candidates with SE-AN were not married (71.4%) and had no children (78.6%). However, 86% of the patients lived with someone: six lived with their partner/husband, and six with the family of origin.

Almost all of the patients had a job during their lifetime (78.6%), including seven employees (who carried out office work, without any specific responsibility), two hairdressers, one bartender, and one lawyer. Among those who had never worked, two were University students. Therefore, only one out of 14 patients never worked or pursued a college career. Among the 11 individuals who had had a job, however, only three people were known to be employed at the time of the analysis (27.3%). For two people, no data regarding their working life were available on their medical records.

Among the 14 patients, seven (50%) had a cigarette smoking habit, while two (14.2%) had a history of alcohol abuse. Three (21.4%) had a history of self-injury.

### Clinical characteristics

The average age of onset of ED symptoms in our sample was 22.7 years (range 11–47), while the average age at which a diagnosis of AN was made in public services (Psychiatry or Dietetics) was 31.5 years (range 16–51). The patients had a mean disease duration of 17 years (range 8–31) combined, in the vast majority of cases (85.7%), with a series of unsuccessful therapeutic attempts. The latency period between the onset of the disease and its recognition by public health services was 8.79 years (range 1–34 years); in nine out of 12 cases (64.3%) the diagnosis was made after at least three years of illness, while in 6 cases (42.9%) after at least seven years.

The most frequent AN subtype in the considered sample was AN-Restrictive (85.7%), while only two patients (14.3%) suffered from the AN-Binge/Purging subtype. The most frequently reported caloric restriction methods were reduced caloric intake and intense physical exercise; this was followed by laxative use, self-induced vomiting, and diuretic use.

The average patient’s Body Mass Index (BMI) reported was 13.43 kg/m^2^ (range 7.53–16.94 kg/m^2^), highlighting the extreme severity of the cases described (according to the DSM-5 [1], AN patients with BMI < 15 kg/m^2^ are classified as showing “extreme severity”). The patient with the lowest BMI included in the case series reached a value of 7.15 kg/m^2^.

Eleven patients (78.6%) had been hospitalized at least once in an internal medicine department because of their ED, due to their severe malnutrition; in addition, among the three patients who had never faced hospitalization, two had previously refused it several times despite the need expressed by their caregivers. In contrast, at least two out of 14 (14.3%) had multiple accesses and one patient was hospitalized at the time of data collection. In parallel, seven of the patients (50%) were admitted at least once to a psychiatric department to manage their disorder. In the examined clinical context, six patients (42.9%) received enteral nutrition through nasogastric tube administration on at least one occasion, while an additional six patients (42.9%) required parenteral nutrition. The purpose of the parenteral nutrition intervention was to augment daily caloric intake and provide supplementary support to oral nutrition exclusively.

Common complications of AN, such as anemia and hypokalemia, and their treatment needs, were also investigated. In 50% of the patients, the occurrence of at least one episode of anemia during the natural history of the disease was reported. Anemia was most commonly macrocytic (57%). Regarding treatment, at least 42.9% of anemia cases were of such severity that they required blood transfusion, 28.6% required only iron and vitamin supplementation. 21.4% had no history of anemia. For the 28.6% no data about the occurrence of anemia as a complication of their disorder was found examining the available clinical records, while several patients refused to take blood tests. At least one episode of hypokalemia was reported in 50% of cases. Of the seven confirmed cases of hypokalemia, 100% required treatment, and at least three were of such severity as to require intravenous infusion therapy.

Other ED complications present within the considered sample, consequences of persistent malnutrition and secondary hormonal disorders typical of AN, included osteoporosis and secondary amenorrhea. In 57.1% of the cases frank osteoporosis was shown and in 28.6% osteopenia was demonstrated. In 93% of patients there was at least one period of secondary amenorrhea during the natural history of the disease (no data regarding one individual); these included two patients taking an Estrogen-Progestin (EP) pill and two who reached menopause before having the diagnosis of AN (one of whom was in early menopause).

The presence of psychiatric comorbidities in the history of these subjects was assessed (Table 4), founding that 100% had at least one other psychiatric diagnosis in comorbidity to the ED (not necessarily present to date); three out of 14 patients (21.4%) had only one psychiatric comorbidity, nine (64.3%) had two, and two patients (14.2%) had up to three psychiatric disorders in addition to AN.

**Table 4:** Types and frequencies of psychiatric comorbidities

6 (57.1%)	MDD
7 (50%)	PD, variably defined:
	One case of Cluster A
	Two cases of Cluster B, including one histrionic and one borderline
	Three cases of Cluster C, including one avoidant and two obsessive-compulsives
	One case not otherwise specified
3 (28.6%)	OCD
3 (28.6%)	Anxiety disorder not otherwise specified
2 (14.2%)	Psychotic disorder
1 (7.1%)	Bipolar disorder
1 (7.1%)	Potomania

*MDD* major depressive disorder, *PD* personality disorder, *OCD* obsessive compulsive disorder

It was reported that 50% of patients had a history of familial psychiatric illness, and 14.2% had a parent with severe obesity.

With respect to the therapeutic approaches used for these patients, previous drug therapy attempts employed in ED treatment (in part related to the management of the various psychiatric comorbidities present in the individual cases), and the execution of ED-specific therapeutic pathways (e.g., Dietary care), were evaluated as far as possible.

Nine patients had experienced at least one ED-specific pathway, while five had never been through one; among the latter, four out of five had rejected the proposed ED treatment course, while one was considering the proposal at the time of the data collection. The setting most frequently used by those who had embarked on an ED treatment course was outpatient (100%), followed by semi-residential and residential (44.4%). Among those who started an ED pathway: six completed it, two dropped out, and one moved away.

Regarding the pharmacological therapies taken by patients during their treatment course, the use of three pharmacological classes mainly used in AN treatment (antidepressants, antipsychotics, and benzodiazepines) was analyzed, also considering the possible combined therapeutic indication with respect to the individuals' psychiatric comorbidities.

Nine out of 14 (64.3%) patients used at least one antipsychotic drug, while 28.6% never used antipsychotics; of one out of 14 patients no data were found about antipsychotic administration from the examined clinical documentations. Among users, at least three used more than one antipsychotic in their history, and the most frequently prescribed drug was olanzapine (66.7%), followed by risperidone (22%) and aripiprazole (22%).

11 of 14 patients (78.6%) used at least one antidepressant drug to manage their psychiatric disorders; two patients never used it (n = 14.3%), and one rejected the suggested treatment. Among antidepressant users, 36.4% used more than one. The most commonly prescribed antidepressant was sertraline (77.8%), followed by venlafaxine (44.4%).

Nine out of 14 individuals (64.3%) used at least one benzodiazepine during their course of treatment in psychiatric services. Four patients (28.6%) never used benzodiazepines: three had not been prescribed and one refused to take them. There was one reported case of benzodiazepine abuse, while for one patient no data was found in the available clinical records regarding sedative medications. The most commonly used benzodiazepine appears to be alprazolam (40%).

Lastly, the information obtained showed that all patients (100%) undertook individual psychotherapy during their treatment process, even though duration, frequency, and type of psychotherapeutic courses were not reported in the records.

## Discussion

The present work gives an insight into SE-AN, analyzing clinical features, treatment approaches, and risk factors that might contribute to the persistence of this disorder. Our patients showed a long history of illness, with an average duration of 17 years, punctuated by therapeutic failures. The majority of patients were diagnosed with the restrictive subtype of AN, characterized by caloric restriction methods, including reduced intake and intense physical exercise. The BMI average was 13.43 kg/m^2^, data highlighting the extreme severity of this clinical sample, aligning with the DSM-5 classification of extreme severity for AN patients with BMI < 15 kg/m^2^ [1]. We identified a high prevalence of hospitalizations due to severe malnutrition or the occurrence of medical complications (i.e., anemia, hypokalemia, osteoporosis, amenorrhea). Moreover, the majority of the sample also suffered from psychiatric comorbidities. The presence and severity of these aspects confirms the condition of intense medical and psychological burden faced by these patients [9, 10].

The treatment history of these individuals has often proven to be complex and ineffective, despite the multitude of approaches used, including outpatient, semi-residential, or residential treatment. In addition, this work has highlighted a difficulty on the part of the health care system in identifying the disease at the time of its presentation, leading to diagnostic delays and higher therapeutic resistance. In fact, consolidation of the symptoms and psychopathological mechanisms over time in AN patients reduces the likelihood of positive outcomes following treatments, consequently limiting the chances of recovery for these individuals [11]. There is growing bio‐behavioral evidence in EDs that the disease changes over time, with maladaptive eating and weight control behaviors becoming more automatic and entrenched [12–17]. Consistent with these results, many clinical studies suggest that response to treatment is more positive in the early stages of the disease (i.e., within the first three years of ED onset), and decreases the longer the condition persists [18, 19]*.*

Likewise, it has been reported that, during early-stage ED, longer disease duration is associated with higher psychological distress and occupational and social impairment [9, 20]*.* Therefore, the lack of—or delay in access to—treatment during the early-stage ED may facilitate chronicity, negatively impact the chances of recovery, impair social and occupational accomplishment [21]. In fact, from an environmental point of view, our sample showed relatively poor social and occupational adjustment, most of the patients not being married (71.4%), without children (78.6%), and unemployed (72.7%) at the time of data collection. These findings are in line with recent studies in the field, which characterizes individuals with SE-AN as impoverished in terms of intimacy and relationships [3, 22], and exhibiting a propensity for economic frugality, some living below the poverty line, without well-remunerated employment behind them [22, 23].

The duration of untreated ED (DUED) is the period of time between disease onset and the start of evidence‐based treatment. In the existing literature, the average DUED is reported to be between two and three years for anorexia nervosa (AN) [24]*.* However, it is noteworthy that our study yielded different findings, as we observed an average DUED of 8.79 years in our sample, with a wide-ranging variation from as short as 1 year to as long as 39 years. This significant deviation from the established averages underscores the heterogeneity and complexity of DUED across different populations, warranting further exploration to elucidate the contributing factors.

DUED can be divided into two distinct stages [25]*.* The initial stage is characterized by delays mostly driven by patient‐related factors, wherein individuals may experience symptoms but fail to recognize the presence of a problem or may not be prepared to seek help. In the second stage, individuals seek treatment, but they encounter service‐level delays, further prolonging the untreated illness period.

Flynn et al. [21] evaluated the role of First Episode Rapid Early Intervention for ED (FREED), finding that FREED, significantly reducing the DUED, is associated with significantly shorter wait times for both assessment and treatment, higher patients compliance to treatment, and possible distress reduction and deterioration prevention. The same results are suggested by Andrés-Pepiñá et al. [26], reporting that a substantial percentage of patients with adolescence-onset AN achieve complete remission of the disorder when they undergo specialist treatment, and an early intervention in AN may help to improve the disorder course. Also, Austin et al. [24] suggested that DUED may be a modifiable factor influencing EDs outcomes and that a shorter DUED may be related to a higher probability of remission.

Diagnostic and treatment delays appear to be partly attributable to gaps in the health system and scarce economic resources, and partly attributable to the ego-syntonic nature of the disorder itself. The treatment refractoriness, pushing the patient away from the therapeutic paths taken, if not rejected out of hand, is usually a result of an incomplete understanding of their disease state [25, 27]. People with AN tend to hide their state of emaciation, avoiding an established relationship with primary care and resorting to emergency departments only when medical problems arise [28], leading in some cases to hospitalization. The prevalence of untreated individuals with EDs is estimated to be as high as 75% [29]. This considerable percentage may be attributed, in part, to comorbid conditions that influence motivation, scheduling constraints, or the need for clinical prioritization within general mental health services. These factors can result in delayed or hindered access to specialized ED services, particularly in cases where individuals with EDs also present with concurrent issues such as self-harm or suicidal behaviors [30].

Diagnostic-therapeutic delays thus lead to high rate of medical comorbidities due to malnutrition, the need for internal medicine/psychiatric hospitalization, as well as the substantial burden of psychiatric comorbidities [9, 10, 20, 31]. Medical comorbidities and complications associated with EDs can range from mild to severe and life-threatening, potentially involving all body systems and placing people at increased risk of medical instability and death [32]. Therefore, understanding how comorbidities and co-occurring medical complications impact EDs is fundamental to treatment and recovery. In addition to the ED-associated medical comorbidities, EDs often occur together with other psychiatric conditions. Psychiatric comorbidities in people with EDs are associated with higher emergency department presentations and hospitalizations and health system costs [33]. Comorbidities may result from symptoms and behaviors associated with the ED, be co-occurring, or precede the ED onset [34, 35]. People with an ED, their caregivers and care providers often face a complex dilemma: the individual with ED needs treatment for not only for their ED but also for their psychiatric comorbidities, and it can be hard to determine which is the clinical priority. This is further complicated because EDs and comorbidities may have a reciprocal relationship of mutual worsening, exacerbating each other's symptoms and negatively impacting treatments and outcomes.

The case series also confirms what literature shows about how the severity of SE-AN cannot be defined solely by BMI value and resistance to treatment [36–38], but also by the multiplicity of possible negative consequences that mark the life course of these patients, and the increasing consolidation of ED related psychopathology [39]. Repeated hospitalizations, severe complications, frequent comorbidities, a variety of unproven drug treatments are all equally present variables that indelibly mark the very long history of the disease.

## Conclusion

This case series presented 14 cases of adult patients affected by SE-AN. It highlighted the relevant issue of resistance to treatment that marks the life course of these subjects, as well as the prospect of a variety of complications, both medical, psychological as well as social. The chronicity of this disorder is determined by the overlapping of numerous elements. First of all, the very nature of the disorder, which often makes the patient less likely to seek treatment, the difficulty of the health system in recognizing the problem in its early stages, but also the presence of an occupational and social impairment. Further studies on the topic are needed to broaden the knowledge of this disorder and its pathogenesis. This will enable us to develop more precise and effective interventions.

## Data Availability

The datasets used and/or analyzed during the current study are available from the corresponding author following a reasonable request.

## Abbreviations

AN: Anorexia Nervosa
BMI: Body Mass Index
DSM-5: Diagnostic and statistical manual of mental disorders, 5th edition
DUED: duration of untreated eating disorder
ED: Eating Disorder
EP: Estrogen-progestin
FREED: First episode rapid early intervention for eating disorders
OCD: Obsessive compulsive disorder
PD: Personality disorder
SE-AN: Severe and Enduring Anorexia Nervosa
